# The Scavenger Function of Liver Sinusoidal Endothelial Cells in Health and Disease

**DOI:** 10.3389/fphys.2021.757469

**Published:** 2021-10-11

**Authors:** Sabin Bhandari, Anett Kristin Larsen, Peter McCourt, Bård Smedsrød, Karen Kristine Sørensen

**Affiliations:** Vascular Biology Research Group, Department of Medical Biology, University of Tromsø (UiT) – The Arctic University of Norway, Tromsø, Norway

**Keywords:** blood clearance, liver, sinusoid, endothelial cell (EC), scavenger receptor, mannose receptor, Fc-gamma receptor IIb, scavenger endothelial cells

## Abstract

The aim of this review is to give an outline of the blood clearance function of the liver sinusoidal endothelial cells (LSECs) in health and disease. Lining the hundreds of millions of hepatic sinusoids in the human liver the LSECs are perfectly located to survey the constituents of the blood. These cells are equipped with high-affinity receptors and an intracellular vesicle transport apparatus, enabling a remarkably efficient machinery for removal of large molecules and nanoparticles from the blood, thus contributing importantly to maintain blood and tissue homeostasis. We describe here central aspects of LSEC signature receptors that enable the cells to recognize and internalize blood-borne waste macromolecules at great speed and high capacity. Notably, this blood clearance system is a silent process, in the sense that it usually neither requires or elicits cell activation or immune responses. Most of our knowledge about LSECs arises from studies in animals, of which mouse and rat make up the great majority, and some species differences relevant for extrapolating from animal models to human are discussed. In the last part of the review, we discuss comparative aspects of the LSEC scavenger functions and specialized scavenger endothelial cells (SECs) in other vascular beds and in different vertebrate classes. In conclusion, the activity of LSECs and other SECs prevent exposure of a great number of waste products to the immune system, and molecules with noxious biological activities are effectively “silenced” by the rapid clearance in LSECs. An undesired consequence of this avid scavenging system is unwanted uptake of nanomedicines and biologics in the cells. As the development of this new generation of therapeutics evolves, there will be a sharp increase in the need to understand the clearance function of LSECs in health and disease. There is still a significant knowledge gap in how the LSEC clearance function is affected in liver disease.

## Introduction

The aim of the present review is to give an outline of the blood clearance function of the mammalian liver sinusoidal endothelial cells (LSECs), which constitute one of the two cellular arms of the hepatic reticuloendothelial system (RES). It is generally accepted today that the hepatic RES consists of two types of specialized clearance cells, namely the liver macrophages, or Kupffer cells, that are geared to take up particles (>200 nm) *via* phagocytosis, and the non-phagocytic LSECs that are specially equipped for clearance of macromolecules and colloids by receptor-mediated endocytosis (Seternes et al., 2002). This understanding is the result of a scientific evolution that has taken place over more than a century, starting with the discovery of the macrophage (Metchnikoff, 1884, 1968), and the use of vital stains to locate the anatomical sites of uptake of blood-borne exogenous and endogenous waste material (Kiyono, 1914; Aschoff, 1924). Uptake of vital stains (a type of colloidal particles) occurred in so-called “reticuloendothelial cells” (Aschoff, 1924), which are endothelial cells with high scavenging activity (Seternes et al., 2002). Readers who wish to look deeper into the historical backdrops and the scientific evolution of the development of the RES concept are referred to Smedsrød (2004) and Sørensen et al. (2012).

A series of experiments during the 1980s established that soluble macromolecules and nanoparticles of various kinds were rapidly cleared from the circulation of mammals mainly by specialized endothelial cells in the liver sinusoids, with negligible uptake in the Kupffer cells (Smedsrød et al., 1990b). Violating the paradigm at the time, that the Kupffer cells alone constituted the RES (Van Furth et al., 1972), these findings came as a surprise. We know today that the LSECs are characterized by a remarkably active receptor-mediated endocytosis making them an important part of the hepatic RES (Smedsrød et al., 1990b; Sørensen et al., 2012).

### Tissue Turnover Processes and Waste Clearance

**FIGURE 1:**
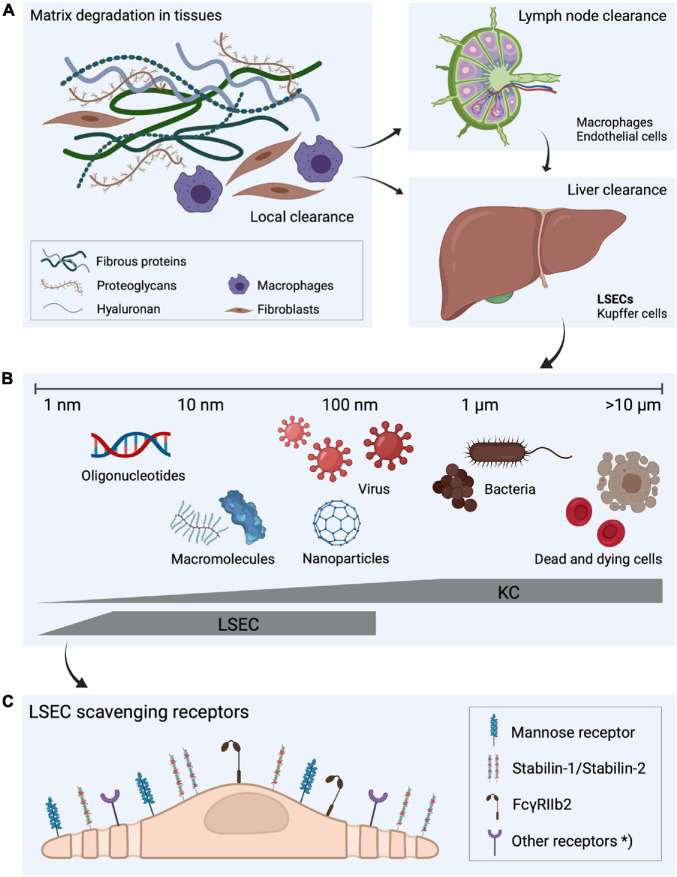
Fate of extracellular matrix turnover products, the dual cell principle of waste clearance and the role of liver scavenger cells in waste clearance. **(A)** Molecular fragments are continuously released during the constant turnover of the extracellular matrix. Some of the degradation products are digested locally but a large proportion is drained to lymph nodes where they are endocytosed by macrophages and sinusoidal endothelial cells (Laurent et al., 1986a; Fraser et al., 1997). The fragments that escape uptake in lymph node cells leak to the blood circulation (Østgaard et al., 1995), and are removed from blood by endocytosis in liver scavenger cells. **(B)** Liver sinusoidal endothelial cells (LSECs) and Kupffer cells, which together make up the largest population of scavenger cells in the body, share the scavenging workload in the liver (Seternes et al., 2002). LSECs are specialized on effective clathrin-mediated endocytosis of soluble macromolecules and nanoparticles, whereas larger particles, such as bacteria and dead and dying cells are cleared by the Kupffer cells, illustrating “the dual cell principle of waste clearance” (Sørensen et al., 2012). **(C)** The uptake of soluble macromolecules in LSECs are mediated by a range of endocytic receptors, with the mannose receptor, stabilin-1, stabilin-2, and FcγRIIb2 being the most investigated. *Other endocytic receptors may also contribute to the effective waste clearance performed by LSECs. Figure created with BioRender.com.

The story about LSECs and other scavenger endothelial cells (SECs) is largely about how the body deals with own and foreign waste products. The metabolic processes in our tissues and cells generate a constant release of all kinds of biological macromolecules. For instance, our connective tissues continuously release considerable amounts of large fragments of matrix macromolecules, such as collagens, procollagen propeptides, and connective tissue polysaccharides, e.g., hyaluronan and chondroitin sulfate proteoglycans. A small portion of these molecules are endocytosed and degraded by local connective tissue cells, whereas the majority are transported with lymph to the lymph nodes, where specialized cells scavenge them (Laurent et al., 1986a; Østgaard et al., 1995; Fraser et al., 1997). The proportion that escapes clearance in lymph nodes are released to the general circulation, where they are finally effectively cleared and degraded by the LSECs (Smedsrød et al., 1985a, 1989, 1990a; Laurent et al., 1986a; Smedsrød, 1988, 1990; Melkko et al., 1994; Østgaard et al., 1995; Malovic et al., 2007; Figure 1 and Table 1). Of note, bone lacks lymph capillaries, and the large amounts of collagen and waste from collagen production that are released from bone tissue are released directly to the blood circulation. Thanks to the LSEC scavenger and mannose receptors these molecules are very effectively removed from the circulation. A different group of waste products that must be removed rapidly from the circulation include the powerful fibrinolytic tissue plasminogen activator (tPA), which is cleared mainly by the LSEC mannose receptor, and to a lesser extent by the galactose receptor of hepatocytes (Smedsrød and Einarsson, 1990). LSECs also participate in elimination of circulating small soluble immune complexes *via* the Fc-gamma receptor IIb2 (FcγRIIb2) (Mousavi et al., 2007). Moreover, macromolecules released from cells under normal or pathophysiological conditions (e.g., lysosomal enzymes and poly- and oligonucleotides) are effectively cleared from the circulation by LSECs (Martin-Armas et al., 2006; Elvevold et al., 2008a) (reviewed in Sørensen et al., 2015). The receptors involved and the speed of clearance observed with several of the waste macromolecules that are eliminated by LSECs are presented in Table 1 and will also be dealt with in more detail in the following sections.

**TABLE 1:** Tissue turnover products cleared from blood mainly by LSECs*, the endocytosis receptor involved in the LSEC uptake, examples of rate of blood clearance of ligands taken up by LSECs following i.v. administration of the ligand, and species examined.

Ligand	LSEC receptor	References	Examples of injected material (dose, inj. site)	Species	Decay of plasma/blood radioactivity (% eliminated)	References
Hyaluronan	Stabilin-2^a^	Smedsrød et al., 1984; McCourt et al., 1999; Zhou et al., 2000; Politz et al., 2002	[^3^H]-hyaluronan (30–32 μg, marginal ear vein)	Rabbit	*t*_1/2_ = 2.5–4.5 min (88% uptake in liver at 19 min after injection)	Fraser et al., 1981
			^125^I-tyramine cellobiose (TC)-labeled hyaluronan (MW = 2.5 × 10^5^) (tail vein)	Rat	*t*_1/2_α = 0.9 min (79% uptake in liver at 30 min after injection)	Dahl et al., 1988
			[^3^H]-hyaluronan (60–130 μg, cubital vein)	Human	*t*_1/2_ = 2.6-5.5min (90% was eliminated from blood after 10 min)	Fraser et al., 1984
Chondroitin sulfate	Stabilin-2^a^	Smedsrød et al., 1985b; Harris and Weigel, 2008	[^3^H]-chondroitin sulfate (CS) and ^125^I-CS proteoglycan	Rat	Clearance rate not examined but the main uptake was in LSECs	Smedsrød et al., 1985b
Heparin	Stabilin-2^b^	Harris et al., 2008, 2009; Øie et al., 2008	^125^I-FITC-labeled unfractionated heparin (0.1 IU/kg, tail vein)	Rat	*t*_1/2_ = 1.71 min (71% was recovered in liver after 15 min)	Øie et al., 2008
Nidogen	SR	Smedsrød et al., 1989	^125^I-TC-nidogen (trace amounts, tail vein)	Rat	*t*_1/2_ = 2-3 min (78% was recovered in liver after 1 h)	Smedsrød et al., 1989
Alpha chains of types I–V and XI collagen	Mannose^c^ receptor	Smedsrød et al., 1985a; Smedsrød, 1990; Malovic et al., 2007	^125^I-FITC-labeled heat-denatured collagen (50 μg, tail vein)	Rat	*t*_1/2_α = 0.8 min (75%) *t*_1/2_β = 3.7 min (25%)	Hellevik et al., 1996
			^125^I-DTAF-collagen (heat-denatured) (0.04 mg/kg, tail vein)	Mouse	*t*_1/2_α = 0.51 min (90.25%) *t*_1/2_β = 36.9 min (9.75%)	Malovic et al., 2007
N-terminal propeptide of types I and III procollagen (PINP and PIIINP)	SR. Stabilin-2	Smedsrød, 1988; Melkko et al., 1994	^125^I-TC-PINP (5 μg, tail vein)	Rat	*t*_1/2_α = 0.59 min (78.5%) *t*_1/2_β = 3.3 min (21.5%)	Melkko et al., 1994
C-terminal propeptide of type I procollagen (PICP)	Mannose receptor	Smedsrød et al., 1990a	^125^I-TC-PICP (10 μg, tail vein)	Rat	*t*_1/2_ = 8.7 min	Smedsrød et al., 1990a
Tissue plasminogen activator (tPA)	Mannose receptor	Smedsrød and Einarsson, 1990	^125^I-tPA (1 μg, tail vein)	Rat	*t*_1/2_α = 0.6 min (65%) *t*_1/2_β = 6.4 min (35%)	Smedsrød and Einarsson, 1990
Lysosomal enzymes	Mannose receptor	Hubbard et al., 1979; Isaksson et al., 1983; Elvevold et al., 2008a	^125^I-cathepsin (10 μg, tail vein)	Mouse	*t*_1/2_α = 0.9 min (63%) *t*_1/2_β = 8.9 min (37%)	Elvevold et al., 2008a
			^125^I-glycosyl asparaginase (trace amounts, tail vein)	Rat	*t*_1/2_α = 0.7 min (63%) *t*_1/2_β = 3.3 min (37%)	Smedsrød and Tollersrud, 1995
			^125^I-α-mannosidase (trace amounts, jugular vein)	Pig	*t*_1/2_ = 5 min (about 60% was recovered in liver, and 18% in lung after 1 h)	Nedredal et al., 2003
Formaldehyde-treated serum albumin (FSA)^*d*^	SR. Stabilin-1, and stabilin2	Blomhoff et al., 1984; McCourt et al., 1999; Li et al., 2011	^125^I-FSA (0.1 mg, femoral vein)	Rat	70% was recovered in LSECs 12 min post injection	Blomhoff et al., 1984
			^125^I-FSA (2 μg, tail vein)	Mouse	*t*_1/2_ = approximately 1–2 min^*e*^ (77% was recovered in liver after 10 min)	Elvevold et al., 2008a
			^125^I-TC-FSA (trace amounts, jugular vein)	Pig	*t*_1/2_ = 2 min (about 53% was recovered in liver, and 26% in lung after 1 h)	Nedredal et al., 2003

*^∗^A more complete overview of macromolecular ligands removed from the circulation by LSECs is presented in Sørensen et al. (2012, 2015). SR, scavenger receptor.*

*^a^The receptor was named the hyaluronan receptor until 1999 when it was found that scavenger receptor (SR) ligands and hyaluronan (HA) bound to the same receptor on LSECs (McCourt et al., 1999). The receptor was later named stabilin-2 (Politz et al., 2002) [aka HARE (Zhou et al., 2000), FEEL-2 (Tamura et al., 2003)].*

*^b^Heparin was found to be a ligand for human stabilin-2 in Harris et al. (2008), and an antibody to the receptor partly inhibited binding of heparin in rat LSECs (Harris et al., 2009) whereas another study in rat did not find heparin binding to stabilin-2 (Øie et al., 2008).*

*^c^Uptake of collagen alpha-chains was until 2007 thought to occur *via* a distinct collagen receptor in LSECs. In 2007 it was found that this receptor was identical to the mannose receptor (Malovic et al., 2007). Binding of denatured collagen/collagen alpha-chains occur *via* the fibronectin type II domain in this receptor (Martinez-Pomares et al., 2006; Napper et al., 2006).*

*^d^FSA is a non-physiological ligand that is much used in studies of LSEC function. It is an SR ligand and is internalized both *via* stabilin-1 and stabilin-2 in LSECs (McCourt et al., 1999; Li et al., 2011).*

*^e^The rate of elimination of ^125^I-FSA from blood was not calculated in Elvevold et al. (2008a) but from the decay curve in Figure 5 of that reference we have estimated that that about 50% of the radioactivity in blood at 1 min post injection was eliminated after 2 min and more than 80% was eliminated after 5 min.*

### Clearance of Virus and Other Nanoparticles From the Circulation

In addition to their significant function of removing endogenous waste material, LSECs also play a role in blood clearance of exogenous ligands such as virus and other nanoparticles. Studies challenging mice with intravenous administration of adenovirus (Ganesan et al., 2011), BK and JC polyomavirus-like particles (VLPs) (Simon-Santamaria et al., 2014) and human immunodeficiency virus (HIV)-VLPs (Mates et al., 2017) showed a rapid and efficient clearance from blood with liver being the main responsible organ and with high uptake in LSECs. Liver was also found to be the main organ for clearing simian immunodeficiency virus in Rhesus monkeys (Zhang et al., 1999). The hepatic clearance was predominantly in LSECs with approximately 90% of eliminated blood-borne adenovirus or HIV-VLPs associated with this cell type, while the remaining associated with Kupffer cells (Ganesan et al., 2011; Mates et al., 2017). Mates and coworkers calculated that the liver sinusoids possessed an astonishing clearance rate of more than 100 million HIV-VLPs per minute (Mates et al., 2017). *In vitro* experiments have also shown that rat LSECs endocytose and degrade T4 bacteriophages (Øie et al., 2020). This efficient viral uptake suggests that LSECs may have an important role in the innate immune defense against viral infections. The receptors responsible for viral endocytosis in LSECs are not yet identified. Other receptors expressed by LSECs (L-SIGN, liver/lymph node-specific ICAM-3 grabbing non-integrin; and LSECtin, liver and lymph node sinusoidal endothelial cell C-type lectin) have been shown to interact with surface glycoproteins of Ebola virus, HIV, SARS coronavirus (CoV), and hepatitis C virus (HCV) (Shetty et al., 2018), and recently with SARS-CoV-2 (Kondo et al., 2021). The function of these receptors in LSECs is however, not well known.

#### Liver Sinusoidal Endothelial Cell Clearance as a Challenge to Delivery of Nanopharmaceuticals

As outlined in Sørensen et al. (2012) and Figure 1 LSECs are geared to take up and metabolize several types of macromolecules and nano sized material <200 nm, a size range that includes most types of nanotherapeutics. Although critical for homeostasis maintenance, the powerful capability of LSECs to remove own and foreign substances from the circulation poses a serious challenge for the development of large size/nano pharmaceuticals. Thus, targeting LSECs with nano sized material is clearly a physiological default system, and focus is therefore commonly on finding ways to avoid uptake of nanopharmaceuticals in these cells. The last decades have seen a surge in the development of the new generation nano drugs. Although promising, with the potential to remedy diseases (e.g., cancer, viral infections, and genetic disorders) for which no cure presently exists, the successful development of these compounds are hampered by the lack of understanding of how to achieve control over the hepatic uptake. It is not possible to cover all aspects of the field in this short paragraph. The use of nanoparticles as carriers of RNA therapeutics, and the challenge of controlling liver uptake can serve as an example. For more literature on nanoparticles that are taken up in LSECs, the reader is referred to Kamps et al. (1997), Sigfridsson et al. (2017), Campbell et al. (2018), Hunt et al. (2018).

One reason for using nanocarriers is to protect RNA therapeutics from being degraded by blood plasma RNases following their intravenous administration. Although chemical modifications of oligonucleotides have been developed to make them resistant to degradation in plasma, the problem of uncontrolled LSEC uptake still exists (Godfrey et al., 2017; Shen and Corey, 2018). Renal filtration also contributes importantly by efficient filtration of material smaller than 6 nm (Choi et al., 2007). In addition, uncontrolled accumulation of these compounds may result in hepatotoxic reactions (Godfrey et al., 2017). Hence, siRNA for silencing of gene expression, or mRNA for gene expression are loaded in nanoparticles to carry these oligonucleotides past the LSECs and the liver and bring them intact to the cellular site of their intended therapeutic activity. Much effort is therefore spent to generate nanoparticles that carry therapeutic RNA to the intended cellular site. Out of a plethora of different types of nanoparticles that have been previously tested as vehicles for therapeutic RNA and other drug candidates, it appears that specially designed lipid nanoparticles have particularly attractive properties. This includes ease of manufacture, reduced immune responses, multidosing capabilities, larger payloads, and flexibility of design (Kulkarni et al., 2018). Although much effort is directed toward designing nanoparticles that reach the intended target cells with high precision and enable the RNA cargo to enter the intracellular compartment, the true “elephant in the room,” that is uncontrolled clearance by LSECs, is still a serious challenge that must be overcome. A few of those nanoparticle-carried RNA therapeutics that have made it successfully to the market include gene correction drugs that target the hepatocytes (Roberts et al., 2020). The LSECs allow passage of these nanoparticles (50 nm) through their fenestrae (i.e., open pores of diameter 100–150 nm). Circulating ApoB binds to these lipid nanoparticles, which mediate binding to the hepatocyte low density lipoprotein (LDL) receptor (Akinc et al., 2010). The same authors showed that conjugation of the particle surface with N-acetylgalactosamine (GalNAc), a ligand for the GalNAc receptor [aka asialoglycoprotein receptor, or Ashwell-Morell receptor, (Morell et al., 1971)] that are present on hepatocytes, but not on LSECs, strengthened the uptake of these lipid nanoparticles to the hepatocytes. Despite the success in using lipid nanoparticles as vehicles for transfer of RNA therapeutics to hepatocytes, the difficulty in achieving efficient delivery to target organs and tissues other than the liver is still a major obstacle preventing widespread usage of oligonucleotide therapeutics. One of the keys to solve this problem would be more precise knowledge on how to avoid unwanted uptake in LSECs.

### Factors Contributing to the Effective Blood Clearance Activity in Liver Sinusoidal Endothelial Cells

Nowadays it is widely appreciated that blood clearance is a central physiological function of LSECs. Moreover, there is general agreement that special endocytosis receptors endow LSECs with their scavenger function. Of note, several additional factors must be taken into consideration to explain the role of LSECs as major blood clearance cells (Table 2).

**TABLE 2:** Factors contributing to the remarkably effective blood clearance activity of LSECs.

***Factors concerning the LSECs proper:***
• Expression of dedicated waste clearing receptors with high receptor ligand affinity
• Extremely fast shuttling (recycling) time of clearance receptors between the cell surface and the early endosomal compartment
• Well-developed apparatus for intracellular trafficking and degradation of endocytosed cargo
• Content of endocytic organelles higher than in most other cell types
***Anatomical and physiological considerations:***
• Strategically located for optimal possibility to survey the blood
• Large total surface facing the blood
• Slow sinusoidal blood flow that allows optimal chance for ligands to encounter clearance receptors

The anatomical location clearly plays a role: lining the hundreds of millions of liver sinusoids and covering a total area of approximately 210 m^2^, i.e., nearly that of a tennis court [Sørensen and Smedsrød (2020); calculated from Blouin et al. (1977)], the LSECs of the human liver are optimally located to effectively survey the large amount of blood that passes every minute. LSECs further make up the largest part of the liver sinusoidal cells, outnumbering the Kupffer cells by about a factor of 2.5 (Pertoft and Smedsrød, 1987).

In addition, a physiological factor contributing to effective interaction of LSECs with the blood is the reduced blood flow through the sinusoids, giving the LSEC clearance receptors ample possibility to remove blood-borne waste macromolecules and colloids that are incompatible with homeostasis. Not only is the sinusoidal blood flow velocity slow, the flow in individual sinusoids is characterized by temporal heterogeneity, which differs between the sinusoidal zones (MacPhee et al., 1995). The intermittence of sinusoidal blood flow varies from fast, slow, stopped, or even reversed. These different flow conditions create very different microenvironments for the liver cells, including LSECs, in zone 1 vs. zone 3. This temporal zonal flow fluctuation, which offers greatly different opportunities for LSECs to survey and bind blood-borne waste macromolecules, needs to be further studied to learn more about the regulation of the clearance activity along the hepatic sinusoid.

Several studies have been published on the expression and ligand specificity of the special LSEC endocytosis receptors, some of which are sufficiently unique to be used as LSEC specific markers at both mRNA and protein levels (Sørensen et al., 2015; Pandey et al., 2020; Sørensen and Smedsrød, 2020). When the goal is to study the LSEC role as blood clearance cells, it appears that not only anatomical aspects and the receptor expression and specificity must be included; the entire endocytic pathway in LSECs must be explored. A literature survey on this topic reveals that major cell physiological events spanning from receptor-mediated ligand internalization to lysosomal ligand processing, are more active in LSECs than in other liver cells and endothelial cells. First, the mode of endocytosis reported for ligands taken up *via* LSEC scavenger and mannose receptors is *via* the clathrin-mediated pathway (Smedsrød et al., 1988; Eskild et al., 1989; Esbach et al., 1994; Hellevik et al., 1998; Kjeken et al., 2001; Hansen et al., 2005). Soluble immune complexes are also internalized *via* clathrin-coated pits after binding to the LSEC FcγRIIb2 (Mousavi et al., 2007). This distinguishes LSECs as a unique member of the family of endothelial cells, since it is generally held that caveolae-mediated endocytosis is a characteristic of endothelial cells. LSECs express caveolin-1 (Yamazaki et al., 2013) but endocytosis *via* caveolae has not been described, and fluid-phase endocytosis is also of little importance for the scavenger function of LSECs (Kjeken et al., 2001).

**FIGURE 2:**
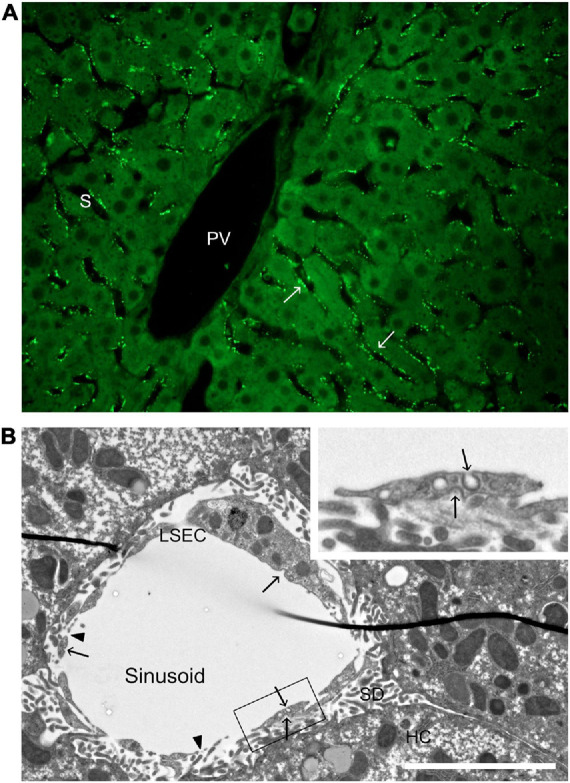
Distribution of a soluble scavenger receptor ligand in the hepatic lobule, and ultrastructure of a liver sinusoid. **(A)** Uptake of FITC-FSA (formaldehyde-treated serum albumin) in mouse liver, 10 min after intravenous administration (dose 2 μg/g bodyweight). Arrows points to FITC-FSA (bright green) located along the sinusoids (S), in a pattern typical of uptake in LSECs. PV, portal vein. **(B)** Transmission electron micrograph of a rat liver sinusoid. The inserted image is a magnification of part of the LSEC in the main image. Arrows point to coated pits and arrow heads to fenestrae. LSEC, liver sinusoidal endothelial cell; SD, space of Disse; HC, hepatocyte. Scale bar 5 μm.

Abundance of clathrin-coated pits and vesicles has been reported repeatedly in LSECs (Wisse, 1970, 1972; Kjeken et al., 2001; Falkowska-Hansen et al., 2007). These were described as “bristle-coated pits and vesicles” in the early, epoch-forming ultrastructural studies of LSECs by Wisse (1970, 1972); clathrin was first described by Pearse (1976). Morphometric analyses of rat liver showed that the density of coated pits at the plasma membrane was about twice as high in LSECs compared to Kupffer cells and hepatocytes (Kjeken et al., 2001). LSECs are highly porous cells with open fenestrae allowing direct passage of plasma proteins and lipoproteins to the subendothelial space of Disse (Wisse, 1970; Wisse et al., 1985; Fraser et al., 1995). The observation that coated pits are present both on the abluminal and adluminal aspects of the sinusoidal lining (Figure 2; Sørensen et al., 2012, 2015), although more abundant toward the sinusoidal lumen, indicates that endocytosis can take place on both sides of the LSEC *in vivo* allowing capture also of filtrated ligands.

Receptors that internalize ligands *via* the clathrin pathway recycle to the cell surface. The half-life for internalization of receptor-ligand complexes is reported to be 17 and 10 s, for LSEC-mediated endocytosis *via* scavenger receptors (SRs; Eskild et al., 1989) and the mannose receptor (Magnusson and Berg, 1989), respectively. This is about 15–35 times as fast as internalization of ligand *via* the galactose receptor of hepatocytes [calculated from table 2 in Eskild et al. (1989)]. This very rapid receptor recycling in LSECs additionally explains the extremely effective clearance of ligands following intravenous administration. Similarly, *in vivo* the circulatory half-life of the ligands removed from blood *via* LSEC receptors are only a few minutes (Table 1).

Following receptor-mediated delivery of ligand to early endosomes, the ligands are transported along the endocytic pathway to the lysosomes for degradation. It is worthy of note that LSECs express very high amounts of Rab5, Rab7, clathrin, α-adaptin, β-adaptin, and rabaptin-5 (Juvet et al., 1997), which are all involved in this pathway. Comparison of the rat LSEC and Kupffer cell transcriptome and proteome further showed higher expression of genes associated with endocytic function, vesicle transport, and positive regulators of endocytosis in LSECs (Bhandari et al., 2020). This adds to the observations that LSECs are highly specialized to perform rapid endocytosis. Additional aspects supporting the notion of LSECs as specialized, professional scavenger cells, is the observations that the cells contain high amounts of lysosomes. Although the LSECs make up only 3.3% of the total liver cell volume, the cells contain impressively 45% of the organ’s endocytic vesicles and 17% of the lysosomal volume (Blouin et al., 1977). Yet another factor contributing to the efficient scavenging activity of LSECs is the specific activity of several lysosomal enzymes which is higher in LSECs than in hepatocytes and Kupffer cells (Knook and Sleyster, 1980; Elvevold et al., 2008a).

In the following sections, we will focus on the major endocytosis receptors of LSECs and the ligands that they remove from the circulation. In addition, we will include information about zonation of receptor expression, species differences, and known changes in receptor expression and clearance function in disease. Finally, we include a section on comparative aspects of clearance function of LSEC-like cells in other organs, and in non-mammalian species.

## Scavenging Receptors in Liver Sinusoidal Endothelial Cells

Liver sinusoidal endothelial cells express a wide range of endocytosis receptors, recently reviewed by Pandey et al. (2020) in this review series. Of these, the main receptors involved in clearance of waste molecules produced in normal turnover processes and disease include stabilin-1 and stabilin-2 (belonging to the LSEC SRs), the FcγRIIb2, and the mannose receptor (Sørensen et al., 2012, 2015).

### Liver Sinusoidal Endothelial Cell Scavenger Receptors

The term “scavenger receptor” (SR) originally described a macrophage receptor which mediates the endocytosis of a broad range of polyanionic molecules (Goldstein et al., 1979). However, this definition needs some refinement as several new SRs and their ligand specificities have been characterized since the definition was first launched. The wide range of ligands to which SRs bind, include: (i) chemically modified proteins such as acetylated and oxidized lipoproteins, maleylated bovine serum albumin (m-BSA), and formaldehyde-treated serum albumin (FSA); (ii) certain polysaccharides such as dextran sulfate; (iii) advanced glycation end-product (AGE) proteins; (iv) amino terminal procollagen propeptides; (v) four stranded, but not one or two stranded, polynucleotides such as poly-inosinic acid and poly-guanylic acid; and other ligands such as anionic lipids on the surface of damaged or apoptotic cells, endotoxin and lipoteichoic acid on pathogenic microorganisms, and crocidolite asbestos (Brown and Goldstein, 1983; Nagelkerke et al., 1983; Blomhoff et al., 1984; Krieger et al., 1993; Krieger and Herz, 1994; Melkko et al., 1994; Smedsrød et al., 1997; Yamada et al., 1998).

The physiological role of SRs is to clean up cellular debris and serve as a part of host defense, but they also play a pathophysiological role in, for example, the accumulation of oxidized LDL (oxLDL) in macrophages leading to the formation of foam cells in atherosclerosis. However, acetylated LDL (acLDL), which does not occur naturally, is a commonly used ligand in the study of SRs. Dextran sulfate is another non-endogenous polyanion used in the study of SRs. This ligand does not discriminate between SRs and mannose receptors, and is therefore regarded as a nonspecific inhibitor of receptor-mediated endocytic pathways (Jansen et al., 1991).

Liver sinusoidal endothelial cells possess significant SR activity responsible for clearing AGE-proteins (Smedsrød et al., 1997; Hansen et al., 2002b), oxLDL (Van Berkel et al., 1991), acLDL (Nagelkerke et al., 1983), hyaluronan (Eriksson et al., 1983; Smedsrød et al., 1984), chondroitin sulfate (Smedsrød et al., 1985b), amino-terminal procollagen propeptides (Smedsrød, 1988; Melkko et al., 1994), nidogen (Smedsrød et al., 1989), and FSA (Blomhoff et al., 1984) from the circulation. FSA is a well-established model ligand used to assess SR activity in LSECs (Figure 2), as well as determining identity and purity of LSEC preparations (McCourt et al., 1999; Sørensen et al., 2015; DeLeve and Maretti-Mira, 2017). This LSEC SR activity is independent of that attributed to the macrophage scavenger receptor (MSR1, aka SR-A1), which is also expressed in LSECs (Hansen et al., 2002a).

The SRs are a growing family [currently 12 different classes (Alquraini and El Khoury, 2020)] of structurally unrelated proteins that have a common affinity for polyanionic molecules. The nomenclature follows the classification defined in PrabhuDas et al. (2017), namely SR-A to SR-L. Of these, LSECs express receptors belonging to class SR-A, SR-B, SR-E, SR-H, SR-J, SR-K, and SR-L (reviewed in Pandey et al., 2020). Despite the expression of several SR subclasses on LSECs, the main work-horse SR on this cell type appears to be SR-H2/stabilin-2, possibly together with SR-H1/stabilin-1 (McCourt et al., 1999; Sørensen et al., 2012). It remains to be determined if the SR-E members LOX-1 and the mannose receptor on LSECs have a role in clearance of the “classical polyanionic” SR ligands. However, the LSEC mannose receptor clearly plays an important role in the clearance of circulating collagen alpha chains (Malovic et al., 2007), C-terminal propeptide of type-1 procollagen (Smedsrød et al., 1990a), tPA (Smedsrød and Einarsson, 1990), and lysosomal enzymes (Elvevold et al., 2008a) (discussed in section “The Mannose Receptor”).

An important difference between human and rodent LSECs regarding SR expression is that CD36 (SCARB3) is widely expressed in human LSECs, and can thus be used as a marker for these cells in tissue sections (Strauss et al., 2017). However, comparative transcriptomic and proteomic profiling of (Sprague Dawley) rat LSECs and Kupffer cells revealed very low CD36 expression in LSECs compared to Kupffer cells (Bhandari et al., 2020), as was also reported in (Li et al., 2011).

The identification and characterization of SRs involved in blood clearance in the LSEC has been a long and winding road in part due to the belief that the LSEC hyaluronan receptor and the receptor referred to as “the LSEC scavenger receptor” were two separate entities. This issue was finally resolved in 1999 when the hyaluronan receptor and a SR on LSECs were found to be one and the same (McCourt et al., 1999), although there was already an indirect suggestion this was the case in 1986 when chondroitin sulfate (a ligand for the hyaluronan receptor) partially inhibited the uptake of a SR ligand (Eskild et al., 1986).

Hyaluronan is a widely distributed negatively charged polysaccharide, first isolated from the vitreous humor (Meyer and Palmer, 1934). It has been attributed with many biological functions such as space filling and joint lubrication, as well as other more specific effects on cell function. Fraser et al. (1981) reported the fate of hyaluronan injected into the blood of rabbits, using ^3^H-hyaluronan, which was labeled on acetyl groups. After 19 min, 88% of the label was detected in the liver, where it was found almost entirely in the non-parenchymal cell (NPC) fraction after Percoll fractionation of liver cells. Some radiolabel was also found in the spleen. The only metabolite detected in the blood or urine was ^3^H_2_O, suggesting complete degradation of the polysaccharide. A subsequent whole body study of the distribution of radioactivity in mice injected intravenously with ^14^C-hyaluronan showed that the polysaccharide was taken up by liver, spleen, bone marrow, and lymph nodes (Fraser et al., 1983).

Eriksson et al. (1983) demonstrated that LSECs, and not Kupffer cells, were the main sites of uptake of hyaluronan by the liver. Smedsrød et al. (1984) performed further studies with primary cultures of parenchymal cells and NPCs to test their ability to bind hyaluronan (at 4°C) and internalize and degrade the ligand (at 37°C), and confirmed that LSECs (and not Kupffer cells or hepatocytes) were able to bind hyaluronan with high specificity and affinity. It was shown that the rates of hyaluronan uptake were highest in LSEC cultures, with degradation products appearing in the supernatant within 30 min of addition of ^3^H-hyaluronan; steady state levels of internalized ^3^H-hyaluronan and degradation products occurred 60–75 min into the incubation. The above results were confirmed *in vivo* with whole body autoradiography studies determining the fate of ^3^H-hyaluronan 10 min after injection into rats; approximately 90% of the injected radioactivity was found in the cytoplasm of LSECs, while none was found in Kupffer cells (Fraser et al., 1985).

The avidity of the endocytic hyaluronan receptor for its ligand increases with the length of the polysaccharide; the dissociation constant ranges from 1.4 μM for octasaccharides to 9 pM for hyaluronan of 6.4 × 10^6^ Da (Laurent et al., 1986b). The smallest hyaluronan fragment that can bind is a hexasaccharide (Smedsrød et al., 1984). The rat receptor also has a threefold greater affinity for chondroitin sulfate than for hyaluronan of the same chain length, but had no affinity for heparin or heparan sulfate (Smedsrød et al., 1984; Laurent et al., 1986b). Chondroitin sulfate, as free chains and as proteoglycan and, to a lesser extent, dermatan sulfate can inhibit the uptake and binding of hyaluronan by LSECs (Smedsrød et al., 1984). Dextran sulfate, a synthetic polysaccharide not found in nature, can also inhibit the binding by LSECs (Raja et al., 1988; McGary et al., 1989).

Studies of digitonin permeabilized LSECs in suspension and culture revealed that 50–75% of the hyaluronan binding sites were intracellular (Raja et al., 1988). The hyaluronan receptors are not degraded after internalization and replaced by newly synthetized receptors, as cycloheximide, an inhibitor of protein synthesis, had no effect on the endocytosis of hyaluronan by cultured LSECs. Instead the receptors are recycled during the continuous endocytosis of hyaluronan, proposed to be *via* a coated pit pathway (McGary et al., 1989).

The “fusion” of the LSEC hyaluronan receptor and LSEC SR activities resulted from a fortuitous discovery by McCourt et al. (1999). The LSEC hyaluronan receptor had previously been wrongly identified as ICAM-1 (McCourt and Gustafson, 1997), so a new attempt was made to purify both the LSEC hyaluronan receptor and the LSEC SR simultaneously from the same LSEC extract. The authors found instead that a Sepharose affinity column coupled with an SR ligand (amino terminal pro-peptides of type I procollagen, PINP) depleted a putative LSEC hyaluronan receptor from ^125^I surface labeled rat LSEC extracts, and vice versa, demonstrating that the LSEC hyaluronan receptor and an LSEC SR were one and the same. A polyclonal antibody to the affinity purified protein blocked LSEC hyaluronan uptake by 80%, and SR ligands by over 50% (McCourt et al., 1999), including AGE-proteins (Hansen et al., 2002b). Amino acid sequence data obtained from the purified rat protein (McCourt et al., 1999) lead to the cloning of the mouse form (Politz et al., 2002). In the latter study, the protein was named stabilin-2 due to its homology to stabilin-1. Both stabilin-1 and stabilin-2 are expressed on LSECs and are constitutively associated with the early endocytic pathway, irrespective of ligand binding (Hansen et al., 2005), but stabilin-1 does not bind hyaluronan (Politz et al., 2002; Prevo et al., 2004).

Stabilin-1 [STAB1, aka FEEL-1 (Tamura et al., 2003), CLEVER-1 (Irjala et al., 2003)], and stabilin-2 [STAB2, aka FEEL-2 (Tamura et al., 2003), HARE (Zhou et al., 2003)] bind a number of other ligands in common, including AGE proteins (Tamura et al., 2003; Hansen et al., 2005) and oxLDL (Li et al., 2011). However, it appears that stabilin-2 has a greater affinity for AGE proteins than stabilin-1 when expressed in CHO (Tamura et al., 2003) and HEK293 (Hansen et al., 2005) cells, while in HEK293 cells stabilin-1 has the greater affinity for mildly oxidized oxLDL and stabilin-2 has the greater affinity for heavily oxidized oxLDL (Li et al., 2011). There are other differences in stabilin-1/2 ligand binding. As mentioned above stabilin-2 (but not stabilin-1) binds hyaluronan (Politz et al., 2002; Prevo et al., 2004), while stabilin-1 (but not stabilin-2) binds SPARC (secreted protein acidic and rich in cysteine) (Kzhyshkowska et al., 2006). Interestingly, human stabilin-2 binds heparin (Harris et al., 2008), while the rat form did not (Smedsrød et al., 1984; Laurent et al., 1986b). Other ligands bound by stabilin-2 include chondroitin sulfates A, C, D, and E, dermatan sulfate and acLDL (Harris and Weigel, 2008). For a more extensive list of ligands bound by stabilin-1 and stabilin-2, see Pandey et al. (2020) in this review series.

Stabilin-2 is specifically expressed in LSECs among liver cells both in rodents and human (McCourt et al., 1999; Politz et al., 2002; Falkowski et al., 2003; Martens et al., 2006; Bhandari et al., 2020) and is a recommended LSEC marker (Geraud et al., 2010; Sørensen et al., 2015; DeLeve and Maretti-Mira, 2017). Immune histochemistry shows staining along the entire length of the hepatic sinusoid in rat (Bhandari et al., 2020), and the receptor is also widely expressed in mouse (Falkowski et al., 2003), and human sinusoids (Martens et al., 2006). In addition to liver, the presence of rat, mouse, and human stabilin-2 is demonstrated in sinusoidal endothelial cells of lymph nodes, spleen, and bone marrow (only studied in mice) (Falkowski et al., 2003; Weigel et al., 2003; Martens et al., 2006; Qian et al., 2009).

Stabilin-1 is expressed in the same organs as stabilin-2, but also in alternatively activated macrophages (M2 phenotype), and the two receptors show a similar staining pattern along the hepatic sinusoid (Politz et al., 2002; Martens et al., 2006). A recent study comparing the transcriptome and proteome of rat LSECs and Kupffer cells confirmed that both stabilin-1 and stabilin-2 were highly specific for LSECs (Bhandari et al., 2020).

#### Liver Sinusoidal Endothelial Cell Scavenger Receptors in Development, Aging, and Disease

The stabilins have an interesting role in development and physiology. During embryogenesis, all endothelial cells in the developing (E13.5) rat liver express stabilin-2, but as the liver develops further, the expression becomes restricted to the sinusoidal endothelium (Yoshida et al., 2007). During aging, there is some reduction in LSEC scavenging, but the level of stabilin-1 and -2 expression in rat LSECs appears to be unchanged regardless of the age of the donor animal (Simon-Santamaria et al., 2010). Despite this age-related reduction in LSEC scavenging, considerable scavenging capacity remained in LSECs from older rats (Simon-Santamaria et al., 2010). Interestingly, in old mice there is reduced endocytosis of stabilin ligands (AGE-BSA) in centrilobular regions of the sinusoid, as observed by *in vivo* microscopy (Ito et al., 2007), and a negative shift in LSEC efficiency of degradation of the AGE proper was observed already in young adult mice compared to prepubertal mice (Svistounov et al., 2013).

In physiology, it was anticipated that the stabilins would be essential for life given their roles in waste clearance. However, stabilin-1 and stabilin-2 knockout mice were phenotypically normal, while stabilin-1/2 double knockout mice exhibited premature mortality and developed severe glomerular fibrosis, while their livers showed only mild perisinusoidal fibrosis without dysfunction (Schledzewski et al., 2011). This would suggest that while the stabilins play a vital role in maintaining health, there is considerable redundancy for their function, possibly mediated by other SRs and hyaluronan receptors. Loss of a single stabilin receptor (either stabilin-1 or stabilin-2) was, however, recently reported to significantly alter the mouse LSEC transcriptome and downregulate some genes (*Coll10*, *Lum*, and *Dec*) coding for carbohydrate binding proteins and defined as potential SRs, suggesting that loss of single receptors may influence LSEC scavenger functions to some extent (Olsavszky et al., 2021).

In certain disease states such as rheumatoid arthritis, osteoarthritis, liver cirrhosis, scleroderma, Werner syndrome, renal failure, psoriasis, and various malignancies the serum level of hyaluronan is elevated (Laurent et al., 1996). This is due either to overproduction of hyaluronan [e.g., in rheumatoid arthritis (Engström-Laurent and Hällgren, 1985), scleroderma (Engström-Laurent et al., 1985a), or psoriasis (Lundin et al., 1985)] or to impaired clearance from the blood [e.g., in liver cirrhosis (Engström-Laurent et al., 1985b)]. In the case of one malignancy, Wilms’ tumor, the overproduction of hyaluronan is so great that it causes the blood to become overly viscous (Tomasi et al., 1966; Wu et al., 1984) as well as causing defects in blood clotting (Bracey et al., 1987). This last example demonstrates the consequences of excessive levels of hyaluronan in the circulation, and therefore the importance of its removal by the LSEC stabilin-2.

### The Fc-Gamma Receptor IIb2

Liver sinusoidal endothelial cells express the endocytic FcγRIIb2 (CD32b) and are the main carriers of this receptor in liver (Mousavi et al., 2007; Ganesan et al., 2012). The FcγRIIb2 is an inhibitory FcγR and mediates endocytosis of small soluble immune complexes. These are formed in the blood circulation when either antibody or antigen is present in excess (Nydegger, 2007), and their clearance in LSECs *via* the FcγRIIb2 provides a way to remove IgG immune complexes without risk of pro-inflammatory activation (Anania et al., 2019). Larger complexes are phagocytosed by Fc receptors expressed on macrophages (Skogh et al., 1985; van der Laan-Klamer et al., 1985, 1986a,b).

The formation of immune complexes is a normal part of the immune defense against soluble antigens. However, deposition of immune complexes in tissues can trigger inflammation and contribute to pathology. Effective elimination is therefore important to preserve homeostasis. The liver is the main organ for clearance of circulating immune complexes (Arend and Mannik, 1971), and uptake of immune complexes in liver was reported more than 60 years ago (Benacerraf et al., 1959). Soluble immune complexes of human serum albumin (HSA) and anti-HSA IgG administered intravenously into rabbits were cleared in liver, with only negligible amounts recovered in lungs, kidney and spleen (Arend and Mannik, 1971). Uptake was independent of circulating complement components, as the tissue distribution was unchanged in complement depleted rabbits and assumed to take place in macrophages. Similar observations were made in mice, and doses known to induce glomerulonephritis could saturate the liver uptake system (Haakenstad and Mannik, 1974).

The first indications that LSECs, and not only macrophages, were involved in immune complex clearance came in the beginning of 1980s, when it was found that freshly isolated rat LSECs plated in serum-free media could avidly bind, but not phagocytose, sheep red blood cells coated with anti-sheep red blood cell IgG (Pulford and Souhami, 1981; Smedsrød et al., 1982). Binding was effectively inhibited by soluble complexes of heat-aggregated IgG and were not dependent on complement, suggesting the expression of FcγRs also in LSECs. Skogh et al. (1985) then reported that radiolabeled large, soluble immune complexes of dinitrophenylated (DNP)-conjugated HSA complexed by IgG distributed to Kupffer cells, whereas smaller complexes of lightly DNP-conjugated HSA complexed with IgG were taken up mainly by LSECs in rats (Skogh et al., 1985). The uptake of large immune complexes in Kupffer cells and small immune complexes in LSECs was also reported by others (van der Laan-Klamer et al., 1985, 1986a,b).

Using peroxidase-anti-peroxidase immune complexes as ligands, Muro et al. (1987, 1988) provided functional evidence of the presence of Fc receptors on Kupffer cells and LSECs both in mouse, rat, and human liver. Immune complexes were equally distributed along the sinusoidal wall, but absent in portal veins and arteries, and in central veins. Interestingly, the immune complexes were found to bind both on the luminal and abluminal aspects of the sinusoidal lining, but more frequently on the luminal side. Also, more binding was observed on LSECs than on Kupffer cells (identified by uptake of 0.5 μM latex beads), and were not present on stellate cells and hepatocytes (Muro et al., 1988). Morphometrical analyses of liver tissue short time after intravenous injection of small-sized BSA/anti-BSA IgG complexes in mice further suggested that LSECs rather than Kupffer cells were the major site for removal of these complexes from the circulation (Kosugi et al., 1992, 1993). However, a substantially higher total uptake in Kupffer cells than in LSECs has also been reported (Johansson et al., 1996). The discrepant findings may depend on the immune complex model system.

LSECs have previously been reported to carry FcγRII and III (Løvdal and Berg, 2001). However, Mousavi et al. (2007) showed by PCR that FcγRIIb2, a splice variant of FcγRIIb, was the only FcγR expressed in rat LSECs. The rat FcγRIIb2 has the same structural and regulatory functions as the mouse receptor and mediates a slow rate of endocytosis. By using an inhibitory antibody to FcγRII/CD32, the authors further proved that FcγRIIb2 was responsible for binding and uptake of soluble immune complexes in rat LSECs. FcγRIIb2 is also the only FcγR in mouse LSECs (Ganesan et al., 2012). The latter study further reported that 72% of total body FcγRIIb2 is expressed in liver, with approximately 90% of the liver receptors in LSECs and 10% in Kupffer cells. The dominating expression of this receptor in liver endothelial cells was also observed in a comprehensive single cell RNA sequencing (scRNA-seq) study which compared the transcriptomes of endothelial cells from 11 mouse tissues (Kalucka et al., 2020).

FcγRIIb has two major forms arising from mRNA splicing (Anania et al., 2019). The difference between the splice variants FcγRIIb1 and FcγRIIb2 is that the cytoplasmic tail of FcγRIIb2 contains a domain needed for accumulation in coated pits, and this domain is disrupted by a 47 amino acid insertion in RIIb1 (Miettinen et al., 1989). Therefore, only FcγRIIb2 can mediate endocytosis and internalization *via* coated pits (Miettinen et al., 1989). In addition to small soluble IgG immune complexes, ligands for the FcγRIIb2 include fibrinogen-like protein 2 (FGL2) (Liu et al., 2008) and measles virus nucleocapsid protein (Ravanel et al., 1997).

The FcγRIIb2 is partly associated with lipid rafts and uses the clathrin pathway for immune complex uptake (Miettinen et al., 1989; Mousavi et al., 2007). In LSECs, internalization *via* FcγRIIb2 is slower than *via* scavenger and mannose receptors (Løvdal et al., 2000; Mousavi et al., 2007), which was partly explained by the association of the receptor with lipid rafts. The FcγRIIb2 is a constitutively recycling receptor and traffics through lysosomal integral membrane protein-II (LIMPII) containing compartments to the LSEC plasma membrane both with and without bound ligand (Mousavi et al., 2007). The intracellular transport of immune complexes to lysosomes in LSECs is slow compared to transport of ligands that are taken up *via* scavenger and mannose receptors (Løvdal et al., 2000) and was suggested to be partly due to repeated recycling of receptor-ligand complexes. An interesting observation was that the kinetics of endocytosis *via* SRs in LSECs was unaffected by the simultaneous uptake of immune complexes, whereas the degradation of immune complexes occurred in the same lysosomes as ligands for SRs (Løvdal et al., 2000).

The distribution of FcγRIIb2 along the hepatic sinusoid shows a different pattern in rodents and human. Immune staining of rat liver sections using the monoclonal SE-1 antibody (Ohmura et al., 1993; Tokairin et al., 2002), which specifically recognizes FcγRIIb2 in rat LSECs (March et al., 2009), showed expression along the entire length of the sinusoid (Tokairin et al., 2002; Bhandari et al., 2020). Similarly in mice, the monoclonal 2.4G2 antibody (Unkeless, 1979), reported to be specific for mouse LSECs in liver sections (Ganesan et al., 2011), stained the entire sinusoidal lining (Ganesan et al., 2012). However, in human liver, immune staining experiments showed low or absent expression of the receptor in the periportal areas (Strauss et al., 2017). This is in accordance with older functional studies showing continuous presence of uptake/binding of immune complexes (interpreted as presence of active Fc receptors) in all sinusoids of rodents, but low or absent binding/uptake close to the portal triad in human liver (Muro et al., 1987, 1988, 1993b).

#### Expression and Role of the Liver Sinusoidal Endothelial Cell FcγRIIb2 in Disease

Containing the immunoreceptor tyrosine-based inhibitory motif (ITIM), FcγRIIb is the only inhibitory Fc receptor and controls many aspects of immune and inflammatory responses. Variations in the *FCGR2B* gene or lack of functional receptor are associated with susceptibility to autoimmune disease, particularly systemic lupus erythematosus (Smith and Clatworthy, 2010). FcγRIIb deficiency also increases the severity of collagen-induced arthritis (Smith and Clatworthy, 2010; increased collagen-specific IgG titres). Furthermore, since 72% of the FcγRIIb2 in mice is in the liver, and 90% of this is in LSECs, it has been speculated that inadequate expression or function of this receptor in LSECs may be a cause of serum sickness and other diseases associated with high levels of soluble immune complexes (Ganesan et al., 2012). Moreover, the high expression of FcγRIIb2 in LSECs, together with studies showing that mice lacking this receptor tend to develop systemic lupus erythematosus (Yajima et al., 2003) is additional evidence that LSECs may play a role in the aetiology of this disease.

Fc-gamma receptors are reported to be downregulated or lost in liver cirrhosis (Muro et al., 1990, 1993b) and in states of proliferation after partial hepatectomy (Muro et al., 1993a), as well as in hepatocellular carcinoma (HCC) (Geraud et al., 2013). A comprehensive single cell transcriptomics study of normal and cirrhotic mouse livers revealed zone specific alterations of LSEC receptor expression in liver cirrhosis induced by CCl_4_ (Su et al., 2021). The study revealed three clusters of LSEC populations corresponding to hepatic zones 1–3. Expression of genes associated with capillarization such as *Cd34*, was most prominent in the pericentral zone (zone 3) in this disease model and was associated with downregulation of *Fcgr2b* (Cd32b) and other receptors. Moreover, the relative share of non-LSEC vascular endothelial cells and lymphatic endothelial cells increased in cirrhotic mice with LSECs constituting 89% of the endothelial cells in normal mouse liver, and 73% in cirrhotic livers. This may lead to decreased immune complex-clearance in LSECs, and rats with CCl_4_-induced liver cirrhosis showed delayed clearance of immune complexes and a weakened reactivity to the ligand in the cirrhotic areas (Muro et al., 1990).

A slight reduction in CD32b expression was noted in aging rat liver but not in human liver (Maeso-Diaz et al., 2018). Interestingly, plasma levels of FGL2, a ligand for FcγRIIb and FcγRIII (Liu et al., 2008) was reported to be elevated in patients with non-alcoholic fatty liver disease (Colak et al., 2011), and in patients with liver cirrhosis and HCC (Sun et al., 2014), suggesting a link to decreased receptor expression.

CD32b, together with stabilin-1, stabilin-2, and lymphatic vessel endothelial hyaluronan receptor-1 (LYVE-1), were sequentially lost during tumor progression in mice with inducible HCC (AST model), as well as in human HCC patients (examined in tissue microarrays) (Geraud et al., 2013). The four LSEC markers were also lost to varying degree in the peritumoral tissue. Interestingly, loss of stabilin-2 and CD32b in the peritumoral tissue of human HCC correlated with significantly increased survival, and the authors suggested that loss of stabilin-2 and CD32b may be markers for subsets of HCC that modify the surrounding microenvironment in a different way.

### The Mannose Receptor

The mannose receptor (MRC1, CD206, or SR-E3), a type I transmembrane protein, is a member of the C-type lectin family and the SR-E family. This receptor is truly a multi-ligand clearance receptor since it has binding affinity for many different ligands in three distinct ligand binding domains. A C-type (Ca^2+^-dependent) carbohydrate binding (aka C-type lectin) domain in eight copies recognizes mannose, N-acetylglucosamine, and L-fucose in the ultimate position of the glycosyl chains of glycoproteins (Ezekowitz et al., 1990; Taylor and Drickamer, 1992; Taylor et al., 1992). A second domain, characterized by a single fibronectin type II repeat, binds specifically to alpha chains of types I–IV collagen (Martinez-Pomares et al., 2006; Napper et al., 2006). A third domain, rich in cysteine, binds with high affinity to sulfated N-acetyl-galactosamine (GalNAc-4-SO_4_) residues (Fiete et al., 1998). The two latter domains do not depend on Ca^2+^ for ligand binding.

The mannose receptor is expressed on macrophage subgroups, perivascular microglia cells and several other cell types, including sinusoidal endothelial cells of liver, spleen, and lymph nodes (Linehan et al., 1999). LSECs are the main carrier of the mannose receptor in the liver of mouse, rat, and pig (Magnusson and Berg, 1989; Elvevold et al., 2004, 2008a; Linehan, 2005; Linehan et al., 2005; Malovic et al., 2007; Bhandari et al., 2020), with lower or absent expression in Kupffer cells (Magnusson and Berg, 1989; Linehan et al., 2005; Elvevold et al., 2008a; Sørensen et al., 2015). Although less explored in human liver, the mannose receptor is reported to be specifically expressed in LSECs along the sinusoids (Martens et al., 2006). Recently, a 30-gene (human) LSEC fingerprint was established based on GFP+ liver endothelial cells from *Tie2*-GFP mice using genes with human orthologs (de Haan et al., 2020). The mannose receptor (*Mrc1*) was ranked top three of the LSEC markers measured by microarray quantification; expression in human liver was confirmed on the protein level. In contrast, scRNA-seq of human liver did not identify *MRC1* amongst the top differentially expressed genes in neither LSECs nor Kupffer cells (MacParland et al., 2018), and a recent bulk proteome and transcriptome profiling comparing rat LSECs and Kupffer cells revealed abundant expression of the mannose receptor (*Mrc1*) in both cells, with the highest expression in LSECs (Bhandari et al., 2020). From the reviewed literature we conclude that the mannose receptor is stably and highly expressed in LSECs in all species examined but that expression in liver macrophages can vary.

Differential expression and distribution patterns along the sinusoids have been described for several LSEC markers in human liver, with immunofluorescence microscopy studies establishing distinct populations of LSECs in periportal and pericentral areas (Strauss et al., 2017). Likewise, scRNA-seq of human liver revealed heterogeneity within different hepatocellular populations, with 806 out of 1,198 expressed genes in LSECs exhibiting significant zonation (Aizarani et al., 2019). However, detailed information about the mannose receptor is not highlighted in these studies. The mannose receptor is not reported to be differentially expressed along the liver sinusoid, and immune histochemical studies indicate uniform expression along sinusoids of mouse and human liver (Martens et al., 2006; Ganesan et al., 2011; Simon-Santamaria et al., 2014). Interestingly, mannose receptor scavenging activity was shown to be zonated in an IL-1β dependent way in mice (Asumendi et al., 1996). In this study, periportally located “Type I” endothelial cells significantly increased their uptake of the mannose receptor ligand ovalbumin following IL-1β treatment compared with “Type II” endothelial cells located close to the central vein.

The mannose receptor is a clearance receptor of high versatility. Several of the ligands recognized by this physiologically important receptor in LSECs is constantly released to the circulation as result of normal tissue turnover processes, and at higher rate during inflammatory episodes. They are then swiftly and silently removed from the blood by LSEC-mediated clearance. The receptor plays an important role in removing collagen fragments from the circulation. Carboxyterminal propeptides of procollagen type I, released during the formation of collagen fibers, are cleared by LSECs after binding to the mannose receptor C-type lectin domain (Smedsrød et al., 1990a). Moreover, free alpha chains of type I collagen, which are released to the circulation as a result of the ongoing connective tissue remodeling of bone and other connective tissues, were reported more than 30 years ago to be removed from the circulation in rat *via* a specific receptor in LSECs (Smedsrød et al., 1985a; Smedsrød, 1990). Receptor-ligand competition studies indicated that this receptor was distinct from other clearance receptors known at the time (Smedsrød et al., 1985a), and it was therefore named the LSEC collagen receptor. However, in 2007 the receptor was found to be identical to the mannose receptor (Malovic et al., 2007), recognizing the collagen type I alpha chains through binding to its fibronectin type II domain. The early LSEC studies further showed that alpha chains of types I, II, III, and IV collagen were internalized *via* the same receptor specificity (Smedsrød, 1989). This is compatible with results obtained from studies using mannose receptor transfection in fibroblasts, revealing that alpha chains of types I, III, and IV collagen bind to the fibronectin type II domain of the mannose receptor (Napper et al., 2006). The binding affinity of free collagen type I alpha chains to LSECs is considerably higher than the affinity to native, triple helical collagen (Smedsrød et al., 1985a; Smedsrød, 1990; Malovic et al., 2007). This makes physiological sense, since the cleavage products from the breakdown of native collagen by vertebrate collagenase, which generates the enzymatic clip that initiates extracellular degradation of native matrix collagen, readily denature at 37°C, and fall apart to free alpha chains (Sakai and Gross, 1967). The result is that free alpha chains, but not native collagen triple helices represent the blood-borne waste products of collagen. Moreover, this receptor binding preference ensures that the LSEC mannose receptor ignores the intact collagen triple helix structures in the space of Disse. It can be calculated that as much as 0.5 g collagen fragments are released daily to the circulation (Ellis, 1961; Christenson, 1997). This illustrates the importance of the LSEC mannose receptor in the clearance of collagen alpha chains from the circulation.

Another example of blood-borne molecules that are cleared by the LSEC mannose receptor is lysosomal enzymes, which contain mannose in terminal position of their glycosylation side chains. These enzymes are initially glycosylated with mannose-6-phosphate residues in the terminal position, which serves as a signal for transfer from the Golgi apparatus to the endosomal/lysosomal compartment. Once inside the lysosomes, acid phosphatase cleaves off the phosphate residues. Hence, when lysosomal enzymes leak out from cells, which takes place both under normal conditions, and at increased rates in inflammation, these molecules are effectively cleared from the circulation by binding to the LSEC mannose receptor (Hubbard et al., 1979; Isaksson et al., 1983; Elvevold et al., 2008b). There are strong indications that the very high specific activity of lysosomal enzymes in LSECs can be partly ascribed to recruitment of these enzymes from the circulation (Elvevold et al., 2008a). This hypothesis is supported by studies in mannose receptor deficient mice showing that LSECs depend on the mannose receptor for recruitment of lysosomal enzymes to maintain normal degradation capacity (Elvevold et al., 2008a).

Tissue plasminogen activator (tPA), a key hemolytic factor, is normally present in the circulation at very low levels. This is mainly due to clearance *via* the mannose receptor in LSECs and to a lesser extent by uptake in hepatocytes (Smedsrød and Einarsson, 1990). This physiologically important mechanism restricts the powerful fibrinolytic activity of tPA to act only at fibrin clots where it binds and performs its enzyme activity by activating the proenzyme plasminogen to fibrinolytic plasmin.

The N-terminal cysteine-rich domain of the mannose receptor recognizes and mediates the clearance of pituitary sulfated glycoprotein hormones, such as lutropin and thyrotropin, from the circulation. This is an important mechanism to control the level of these hormones (Simpson et al., 1999).

#### Role of the Liver Sinusoidal Endothelial Cell Mannose Receptor in Inflammation and Disease

In addition to being responsible for the housekeeping clearance of waste substances, the mannose receptor on LSEC is also involved in the clearance of molecules such as lysosomal enzymes, tPA and myeloperoxidase released during the inflammatory response (Gazi and Martinez-Pomares, 2009). Thus, the mannose receptor contributes to restore homeostasis after inflammatory episodes, a function that links LSECs tightly to the resolution phase of the inflammatory response.

Through its recognition and binding of exogenous molecules such as virus, bacteria and fungi by the C-type lectin domains, the mannose receptor is considered to be an important pattern recognition receptor (PRR) involved in host defense (Stahl and Ezekowitz, 1998). Interestingly, mannose receptor deficiency did not translate into increased susceptibility to infection with *Candida albicans*, *Pneumocystis carinii*, or *Leishmania* spp. in mice (Lee et al., 2003; Swain et al., 2003; Akilov et al., 2007), but variations in the mannose receptor gene (*MRC1*) may be associated with increased susceptibility to chronic inflammatory diseases such as asthma and sarcoidosis in humans (Hattori et al., 2009, 2010). In liver disease, the soluble mannose receptor is used as a macrophage activation marker to predict disease severity and prognosis in conditions such as alcoholic liver disease, primary biliary cholangitis, and Hepatitis B (Sandahl et al., 2017; Li et al., 2019; Bossen et al., 2020).

Due to their anatomical location, LSECs are the first cell type to encounter blood-borne antigens reaching the liver. Hence, it is not surprising that these cells have important innate and adaptive immunological functions (Shetty et al., 2018). In addition to the silent removal of waste molecules, endocytosis of ligands by some SRs, including the mannose receptor, may promote potent pro-inflammatory and anti-inflammatory signaling (Canton et al., 2013). Several receptors highly expressed by LSECs have been shown to interact with different viruses (Lin et al., 2003; Marzi et al., 2004; Gramberg et al., 2005; Lai et al., 2006; Li Y. et al., 2009) and the mannose receptor may mediate dengue virus infection of human macrophages (Miller et al., 2008). Many viruses are highly mannosylated (Zhang et al., 2004), which makes them a likely ligand for the mannose receptor; however, the contribution of the mannose receptor to viral uptake in LSEC is unknown. LSECs can also cross-present antigens to CD8+ T cells by the help of the mannose receptor which takes up, processes and transfers antigen to MHC class I molecules (Limmer et al., 2000; Burgdorf et al., 2007), a process that has been shown to promote CD8+ T cell tolerance in mice (Schurich et al., 2009).

### Other C-Type Lectins and Receptors With Suggested Roles in Liver Sinusoidal Endothelial Cell Blood Clearance

Besides the mannose receptor, LSECs express several other receptors in the c-type lectin family, including L-SIGN (DC-SIGNR and CLEC4M), and LSECtin (CLEC4G) (Bhandari et al., 2020).

In a study comparing the sequenced mRNA transcriptome and proteome of LSECs and Kupffer cells from Sprague Dawley rats, L-SIGN was highly expressed in LSECs only, and low in Kupffer cells (Bhandari et al., 2020). L-SIGN is also strongly and constitutively expressed in human (Pohlmann et al., 2001) and mouse LSECs and can be upregulated in response to treatment with cytokines (Lai et al., 2006). The functional role of the receptor on LSECs is however, not well known, but L-SIGN on other endothelial cells can bind viruses such as HCV (Gardner et al., 2003) and HIV (Pohlmann et al., 2001). Recently, human L-SIGN was shown to act as a receptor for SARS-CoV-2 (Kondo et al., 2021) and the hypothesis was presented that L-SIGN mediated SARS-CoV-2 infection in LSECs, and subsequent activation of the sinusoidal endothelium contributes to COVID-19-associated coagulopathy in patients.

Liver and lymph node sinusoidal endothelial cell C-type lectin is related to L-SIGN and is expressed predominantly by sinusoidal endothelial cells of human liver and lymph nodes (Liu et al., 2004). In a study establishing a 30-gene (human) LSEC signature (de Haan et al., 2020), LSECtin/CLEC4G was ranked as the most highly expressed LSEC marker protein in mouse liver tissue. Expression was also high in rat LSECs compared to Kupffer cells (Bhandari et al., 2020).

High mRNA expression of LSECtin/*CLEC4G*, as well as L-SIGN/*CLEC4M*, has also been shown in human LSECs by single cell sequencing of liver cells (Aizarani et al., 2019). *CLEC4G* was further found on the list of the top 20 most differentially expressed genes in the human liver endothelial cell cluster hypothesized to correspond to “Type-2” LSECs (midzonal and pericentral area), while not appearing on the list of differentially expressed genes in the endothelial cluster corresponding to “Type-1” LSEC (periportal area) (MacParland et al., 2018), indicating a similar zonated pattern as reported for *LYVE1* (Strauss et al., 2017). The LSECtin receptor binds to mannose, N-acetylglucosamine (GlcNAc) and fucose, and has been reported to act as a receptor for different viruses such as the Japanese encephalitis virus (Shimojima et al., 2014), filovirus (Ebola), SARS Coronavirus (Gramberg et al., 2005), Lassa virus (Shimojima et al., 2012) and the lymphocytic choriomeningitis virus glycoprotein (Shimojima and Kawaoka, 2012). The contribution by LSECtin in viral uptake is not well known, but the receptor is potentially involved in the regulation of immune responses toward HCV through interaction with L-SIGN (Li Y. et al., 2009). Although possibly mediating viral uptake, the role of LSECtin in LSEC endocytosis is so far unknown.

Lymphatic vessel endothelial hyaluronan receptor (LYVE-1) is a hyaluronan receptor initially believed to be predominantly located in lymphatic endothelial cells (Banerji et al., 1999; reviewed in Jackson, 2004). Constitutive expression of LYVE-1 is also found in LSECs (Mouta Carreira et al., 2001) and sinusoidal endothelia of human lymph nodes and spleen (Banerji et al., 1999), as well as in vascular endothelial cells of murine lung, adrenal gland, and heart (Zheng et al., 2016) and subsets of tissue macrophages (Schledzewski et al., 2006). Immune labeling of tissue sections show that the distribution of the receptor in human liver is zonated along the sinusoids with LSECs in the periportal area (hepatic zone 1) being negative or low for LYVE-1 while LSECs in midzonal and pericentral areas (hepatic zones 2 and 3) have a high expression of LYVE-1 (Strauss et al., 2017). Differential expression of *LYVE1* in distinct populations of liver endothelial cells was also confirmed by scRNA-seq of human liver cells (MacParland et al., 2018). A zonated expression pattern of LYVE-1 is also reported in mouse liver with the strongest signal observed in the midzonal sinusoids (Mouta Carreira et al., 2001).

Putative functions of LYVE-1 are hyaluronan clearance from the lymph (Prevo et al., 2001) and regulation of leukocyte adhesion and migration within the lymphatic circulation (reviewed in Jackson, 2004). Stabilin-2 is considered the major endocytic receptor for hyaluronan in LSECs (McCourt et al., 1999; Zhou et al., 2000; Harris and Baker, 2020), leaving the relative contribution of LYVE-1 in this process to be unknown. The contribution of LYVE-1 to endocytosis of other endogenous ligands, as well as elimination of foreign particles circulating in the blood, is not fully explored, but mRNA expression of *Lyve1* in murine liver and lung was increased within 4–8 h after LPS-stimulation (Zheng et al., 2016). Endocytosis of 20 nm latex particles by endothelial cells was also increased following LPS-stimulation, but only observed in the lung. LYVE-1 is further suggested to have a role in wound healing and tumor formation (Schledzewski et al., 2006).

The expression of some of these receptors has been reported to be affected by pathological conditions, with LYVE-1 (along with stabilin-1, stabilin-2, and FcγRIIb) being downregulated in human liver cancer (HCC) and cirrhosis (Mouta Carreira et al., 2001; Geraud et al., 2013), and LSECtin being downregulated in HCC (Aizarani et al., 2019).

## Liver Sinusoidal Endothelial Cell Subpopulations and Heterogeneity

An increasing number of studies show spatial heterogeneity of hepatic cells (including hepatocytes, LSECs, hepatic stellate cells, and Kupffer cells) along the porto-central axis (Strauss et al., 2017; Felmlee et al., 2018; Aizarani et al., 2019; Ben-Moshe and Itzkovitz, 2019; Blériot and Ginhoux, 2019; Ma et al., 2020; Koch et al., 2021; Payen et al., 2021). Historically, Wisse et al. (1983) reported an increase in the frequency of fenestrae in LSECs from the portal tract toward the central vein (Wisse et al., 1983). Continual studies on this aspect during the 1990s expanded our knowledge about the differential LSEC response along the sinusoids against various stimuli, substantiating the notion of some functional heterogeneity along the sinusoid (Scoazec et al., 1994; Asumendi et al., 1996; Dini and Carla, 1998). Recently, two LSEC subtypes were reported to exist along the human hepatic sinusoid, based on immune histochemistry of normal human liver, with low or absent expression of CD32 and LYVE-1 periportally (Strauss et al., 2017). The application of single-cell sequencing protocols in addition to conventional methods allows information about tissue complexities (cellular compositions) and cellular heterogeneity, the phenotype of a rare cell population, or the disease-associated cellular phenotype. Recently, several scRNA-seq studies have unraveled the complexity of the liver tissue and comprehensively characterized the hepatic cell types at the molecular level. ScRNA-seq studies have undoubtedly strengthened the evidence and validated the complex labor division among various hepatic cell types, heralding the tremendous spatial heterogeneity and complexity within liver lobules (Halpern et al., 2017, 2018; MacParland et al., 2018; Aizarani et al., 2019; Ben-Moshe et al., 2019; Ramachandran et al., 2019). Studies suggest that more than 50% of the expressed genes within hepatocytes, as well as in LSECs, show zonation (Halpern et al., 2018; Ben-Moshe et al., 2019). So far, few of these gene expressions have been validated with complementary techniques at single cell levels, and functional studies will be needed to understand how differences in gene expression along the sinusoids may affect LSEC scavenger functions.

## Scavenger Endothelial Cells in Other Vascular Beds

The important clearance function of LSECs is well documented (Sørensen et al., 2012). It is noteworthy, however, that specialized endothelial cells exhibiting LSEC-like clearance activity are present also in some organs other than liver. The early vital stain investigators observed accumulation of stains like lithium carmine in several organs in addition to the hepatic RES; ample uptake was reported in the “reticuloendothelium” of spleen, lymph nodes, bone marrow, adrenal cortex, and pituitary anterior gland (Kiyono, 1914; Aschoff, 1924). Although the investigators at the time had no means to accurately identify the RES cells of these organs, the conclusion nearly a century later that intravenously administered lithium carmine is cleared mainly by the LSECs in liver (Kawai et al., 1998), indicates that the cells in other organs that were noted to take up this vital stain, were LSEC-like SECs, in addition to macrophages.

Studies on clearance of physiological waste macromolecules in extra-hepatic RES organs are scarce. In mice, specialized SECs of the bone marrow, which line the sinusoids of this organ, express functional stabilin-1 and stabilin-2, enabling these cells to take up ligands (FSA, AGE-products) that are also avidly taken up *via* these receptors by LSECs (Qian et al., 2009). Likewise, alpha chains of type I collagen, a physiological ligand for the LSEC mannose receptor, were also cleared by the bone marrow SECs, suggesting the presence of both stabilin-1 and -2 and mannose receptors in these cells (Qian et al., 2009). In pig, uptake of FSA and the mannose receptor ligand α-mannosidase were observed in lung endothelium, in addition to uptake in LSECs (Nedredal et al., 2003).

Lymph nodes and spleen, two other extrahepatic organs suggested by the early vital stain scientists as RES members, express several of the same signature clearance receptors as those found in LSECs (Martens et al., 2006). Human lymph node and spleen tissue analyzed by gene profiling and immune histochemistry here demonstrated the presence of stabilin-1, stabilin-2, LYVE-1, and the mannose receptor in sinusoidal endothelial cells of these organs.

Choriocapillaris endothelial cells (CCEs) have recently been implicated as SECs, employing stabilin-2 to clear waste molecules generated in the metabolically active retina (Li R. et al., 2009). It was proposed that CCEs play a significant role in the clearance of AGE products, that – if allowed to accumulate – may contribute to the generation of age-related macular degeneration. The study was done with cells from bovine eyes, and studies in human CCEs is needed to follow up the hypothesis.

### Phylogenetic Aspects – Scavenger Endothelia in Other Vertebrate Classes

**FIGURE 3:**
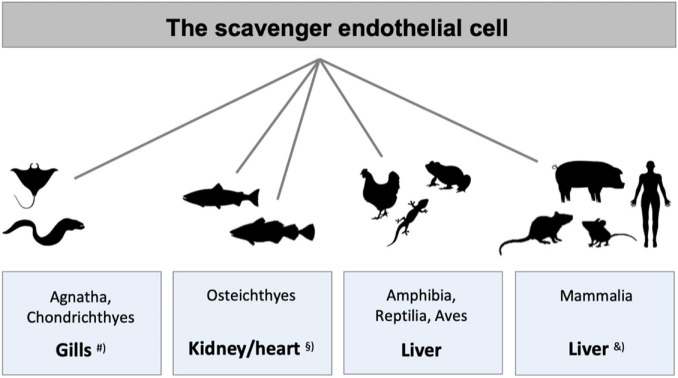
Species differences in the localization of main populations of scavenger endothelial cells (SECs). The figure illustrates the organs that harbor the main populations of specialized SECs in different vertebrate classes. ^#^SECs are localized in special gill arteries in hagfish, lamprey (both Agnatha), and ray (Chondrichthyes) (Seternes et al., 2002). ^§^ In adult bony fish (Osteichthyes) SECs constitute the endothelium of the venous sinusoids in the kidney hematopoietic tissue in crucian carp (Seternes et al., 2002) and salmonid fish (Dannevig et al., 1990, 1994; Smedsrød et al., 1993; Seternes et al., 2002), and the atrial and ventricular endocardium in Atlantic cod (Smedsrød et al., 1995; Sørensen et al., 1997, 1998, 2001; Seternes et al., 2001a, 2002). In all higher vertebrate classes LSECs represent the major SEC population, studied in frog (Seternes et al., 2002), lizard (Seternes et al., 2002), chicken (Seternes et al., 2002), rodents (Smedsrød et al., 1990b; Seternes et al., 2002; Sørensen et al., 2015), and pig (Nedredal et al., 2003; Elvevold et al., 2004). ^&^In addition to the central scavenger function of LSECs in mammals, studies in rabbit and rodents also show scavenging function of the sinusoidal endothelium in spleen, bone marrow, and lymph nodes (Fraser et al., 1983; Qian et al., 2009; Simon-Santamaria et al., 2014), and in pig, scavenging activity is reported in lung endothelium, in addition to LSECs (Nedredal et al., 2003).

The findings by the early vital stain scientists suggested that not only mammals, but also species belonging to the other classes of the vertebrate kingdom, were equipped with a RES that accumulated vital dyes (Kiyono, 1914). However, animal species of phylogenetically older vertebrates displayed a distinct, yet different RES distribution than in the land-based vertebrates. Hypothesizing that this distribution might reflect the distribution of SECs, a study was carried out to investigate if ligands reported to be taken up by LSEC clearance receptors in mammals could be used to determine the distribution of RES in vertebrate classes other than mammals (Seternes et al., 2002). The result of this screening study, summed up in Figure 3, revealed that ligands for the mammalian signature LSEC clearance receptors stabilin-2 and the mannose receptor, were indeed cleared from the circulation in the RES organs reported by the early vital stain scientists. In addition, the finding that particles large enough to be cleared exclusively by phagocytosis accumulated in macrophages, revealed the presence of a pan-vertebrate dual cell principle of blood clearance, with particles >200 nm taken up mainly in macrophages, while macromolecules and colloids <200 nm were cleared mainly by uptake in SECs (Seternes et al., 2002). The ligand distribution screening was performed by recording the anatomical site of ligand uptake following intravenous administration of selected (fluorescence- or radiolabeled) soluble SR and mannose receptor ligands. It is noteworthy that the endocardially located SECs of Atlantic cod (*Gadus morhua*) responsible for the blood clearance of the tested ligands in this species, express stabilin-2, as shown by western blot analysis revealing that lysates from purified cod endocardial endothelial cells, and pig and rat LSECs all reacted with an antibody to whole rat stabilin-2 (Sørensen et al., 2012).

Moreover, recent studies in embryonic zebrafish (*Danio rerio*) showed ample uptake of hyaluronan in SECs located in the caudal vein and vein plexus. This uptake was completely abolished in mutants lacking functional stabilin-2 (Campbell et al., 2018). These findings in the Atlantic cod and zebrafish show that stabilin-2 is well conserved over the considerable phylogenetic time span from bony fishes to mammals. A similarly high degree of phylogenetic conservation is also suggested for the mannose receptor, which is present not only in mammals. It has also been cloned and characterized in the zebrafish (Wong et al., 2009; Zheng et al., 2015). The expression of mannose receptor mRNA was much higher in kidney than in other organs of the zebra fish. Although the role of the zebrafish mannose receptor in the clearance of the same physiological waste molecules as in mammals has not yet been confirmed, the deduced amino acid sequences shared highly conserved structures with the corresponding mammalian receptor and contains a cysteine-rich domain, a single fibronectin type II domain, and eight C-type lectin domains. This strongly indicates that this receptor in the zebrafish serves the same blood clearance function as in mammals.

Stabilin-1 is also expressed in zebrafish and is required for clearance of small (6–30 nm) anionic nanoparticles from the circulation, whereas a combined contribution of stabilin-1 and stabilin-2 is required for clearance of larger (approximately 100 nm) anionic nanoparticles. This finding represents significant information about the influence of the size of anionic nanoparticles for targeting the mammalian LSECs (Arias-Alpizar et al., 2021).

A recent study in 5-day-old zebrafish embryos showed that brain lymphatic endothelial cells (BLECs) play an important role as SECs in the brain, taking up waste substances such as proteins, polysaccharides and virus particles (Huisman et al., 2021). Interestingly, it was found that BLECs and microglia (brain macrophages) work side by side to remove extracellular components from the brain, thus maintaining homeostasis in the brain meninges. In this collaborative function, BLECs, like LSECs and other vertebrate SECs, are particularly active in the clearance of macromolecules and nano particles up to a certain size, whereas the microglia are more active in the uptake of larger material, e.g., bacteria. This collaborating arrangement of the two clearance cells of the brain is another striking example of the vertebrate dual cell principle of waste clearance (Sørensen et al., 2012).

At variance from the observation in mammals that hyaluronan and other waste macromolecules administered subcutaneously or intramuscularly are largely taken up in SECs of local lymph nodes, with only low amounts being cleared by LSECs, radiolabeled hyaluronan injected subcutaneously in the Atlantic cod was taken up mainly in the endocardial SECs (Sørensen et al., 1997). The lack of lymph nodes in fish explains this observation, demonstrating the importance of blood clearance of waste macromolecules in the main SEC organs of these species. Following development of a method for isolation and culture of primary cod endocardial endothelial cells (representing cod SECs) (Koren et al., 1997), studies were carried out *in vitro* to explore in more detail the effective mechanism of the uptake of physiological waste macromolecules in these cells (Koren et al., 1997; Sørensen et al., 1998, 2001; Seternes et al., 2001b). Those studies revealed that the cod SECs endocytose ligands for the scavenger and mannose receptors in the same way as has been demonstrated for mammalian LSECs. Receptor-mediated endocytosis and degradation was responsible for rapid and high-capacity uptake of the physiological molecules hyaluronan (Sørensen et al., 1997), chondroitin sulfate (Seternes et al., 2001b), lysosomal enzymes (Sørensen et al., 2001), N-terminal propeptide of type I procollagen (Sørensen et al., 1998), and collagen alpha chains (Smedsrød et al., 1995; Koren et al., 1997).

## Concluding Remarks

As the result of normal metabolic processes, large amounts of macromolecules from various tissues must be swiftly and silently removed to clean the blood and maintain homeostasis. The LSECs exhibit a remarkably efficient blood clearance capability. This is due largely to their extremely rapid and high-capacity endocytosis, mediated by receptors specifically recognizing a variety of different waste macromolecules. Moreover, the LSECs, lining the hepatic sinusoids, are strategically located for optimal survey of the blood. Equipped with endocytic pattern-recognition receptors that display multi-ligand binding domains, these cells clear a plethora of different types of waste molecules, many of which are DAMPs and PAMPs with the potential to activate immune cells if allowed to circulate. Hence, the waste clearance activity of LSECs represents a silent removal of molecules, maintaining homeostasis.

Our knowledge about the clearance activity of LSECs in various pathophysiological conditions are rudimentary. Questions that need to be answered include establishing how liver is affected by changes in the LSEC scavenger function in various pathophysiological conditions. Moreover, development of the new generation of pharmaceuticals including macromolecular and nanosized compounds are seriously hampered due to undesired clearance of these compounds in LSECs. This is still a major challenge that needs to be solved.

Studies in various mammalian tissues have revealed the presence of SECs with striking functional similarity to the LSECs. Animal species from all vertebrate classes employ SECs to clear waste macromolecules from the circulation, in the same way as LSECs of mammals. However, it is noteworthy that phylogenetically old vertebrate classes (jawless, cartilage, and bony fishes) carry their SECs in organs other than liver. Apart from this difference, the functional similarities of SECs from all vertebrates are prominent, revealing a remarkably well conserved pan-vertebrate waste clearance system that has been well conserved over a considerable phylogenetic time span.

## Author Contributions

All authors substantially contributed to the design and writing of the review. AL: figures. KS: images. KS and BS: editing. All authors approved the final version.

## Conflict of Interest

The authors declare that the research was conducted in the absence of any commercial or financial relationships that could be construed as a potential conflict of interest.

## Publisher’s Note

All claims expressed in this article are solely those of the authors and do not necessarily represent those of their affiliated organizations, or those of the publisher, the editors and the reviewers. Any product that may be evaluated in this article, or claim that may be made by its manufacturer, is not guaranteed or endorsed by the publisher.

## Abbreviations

acLDL: acetylated low density lipoproteins
AGE: advanced glycation end-products
Fc γ RIIb2: Fc-gamma receptor IIb2
FSA: formaldehyde-treated serum albumin
HCC: hepatocellular carcinoma
LSEC: liver sinusoidal endothelial cell
LSECtin: liver and lymph node sinusoidal endothelial cell C-type lectin
L-SIGN: liver/lymph node-specific ICAM-3 grabbing non-integrin
LYVE-1: lymphatic vessel endothelial hyaluronan receptor-1
NPC: non-parenchymal liver cells
oxLDL: oxidized low density lipoprotein
RES: reticuloendothelial system
SEC: scavenger endothelial cell
scRNA-seq: single cell RNA sequencing
SR: scavenger receptor
tPA: tissue plasminogen activator
VLP: virus-like particle.
